# Dairy Heifer Motivation for Access to a Shaded Area

**DOI:** 10.3390/ani11092507

**Published:** 2021-08-26

**Authors:** Clarissa Silva Cardoso, Marina A. G. von Keyserlingk, Luiz Carlos Pinheiro Machado Filho, Maria José Hötzel

**Affiliations:** 1 Laboratório de Etologia Aplicada e Bem-Estar Animal, Departamento de Zootecnia e Desenvolvimento Rural, Universidade Federal de Santa Catarina, Florianópolis 88034-001, Brazil; clarissa.cardoso@gmail.com (C.S.C.); pinheiro.machado@ufsc.br (L.C.P.M.F.); 2 Animal Welfare Program, Faculty of Land and Food Systems, The University of British Columbia, Vancouver, BC V6T 1Z4, Canada; nina@mail.ubc.ca

**Keywords:** operant conditioning, behaviour, heat stress, animal welfare, social rank

## Abstract

**Simple Summary:**

The aim of this study was to determine the motivation of pasture housed dairy heifers to access shade during southern Brazil’s summer and autumn months, and if social rank affected shade use. Heifers valued shade and worked to access it, particularly on hot summer days. Heifers of higher social rank displaced other heifers more often and spent more time in the shaded areas, particularly in the area with trees plus a shade cloth, than the intermediate and subordinate heifers.

**Abstract:**

We used an operant conditioning paradigm to test the motivation of non-pregnant dairy heifers to access shade during the summer and autumn months (January to June) in southern Brazil. Dairy heifers (*n* = 18) were trained to push a weighted gate to access either an experimental area containing both a shaded (simple tree shade and shade cloth) and unshaded area (WITH SHADE) or an experimental area with no shade (BARREN). The latency to push the weighted gate, and the maximum weight pushed by each heifer, were recorded in both the summer and the autumn. Temperature and humidity were recorded continuously for the duration of the study and were used to calculate the heat index. The maximum weight pushed to enter the WITH SHADE area was greater in summer than in autumn, and was inversely related to the latency to push the weighted gate. Heifers refused to work for access to the BARREN environment. As expected, both the maximum ambient temperatures and heat index were higher in summer than in autumn, and also higher in the non-shaded areas than under the shade in both seasons. Heifers of higher social rank displaced other heifers more often, and spent more time in the shaded areas, particularly in the area with trees plus a shade cloth, than the intermediate and subordinate heifers. We conclude that shade is an important and valued resource for heifers reared on pasture-based systems in sub-tropical environments, particularly during the hot summer months.

## 1. Introduction

In many parts of the world, cattle may suffer from heat stress following exposure to solar radiation [1], which can negatively impact both the physiology [2] and the behaviour of cattle [3]. Providing access to shade may mitigate these negative effects [4]. Depriving animals the opportunity to seek shade may also affect their ability to perform natural behaviours, as well as compromise their affective states and their biological functioning [5]. However, little is known about the motivation expressed by cattle to access shade. 

Motivational tests for animals are used in research to investigate the importance of resources to animals [6]. The motivation to access shade can therefore be experimentally tested to measure its importance to cattle. Motivation is defined as the process within the brain controlling which behaviours and physiological changes occur and when they occur [7]. Operant conditioning, where animals are required to perform a particular behaviour to receive a reward [8], is a common experimental paradigm used to assess motivation [9,10]. Briefly, the animals learn to associate the reward with the need to perform a specific behaviour; a measurable cost can then be quantified following the performance of the behaviour, often referred to as the price paid [11]. The underlying assumption is that the more the animals pay to access the reward, the stronger the motivation [10]. Animals pay more for resources they consider to be valuable (inelastic demand) and less for valuable resources that they consider less important (elastic demand) [12]. The use of different weights that must be moved by the animal to gain access to a resource is a common method to measure motivation in several species (e.g., hens [13], rats [14], mink [15], and cattle [16,17,18]). The more weight that is pushed to gain access, the greater the motivation. Using this approach, Schütz et al. [19] showed that lactating dairy cows preferred to stand in the shade rather than lie down in the sun, even after 12 h of lying deprivation, indicating that shade is a highly valued resource.

The aim of the current study was to investigate dairy heifer motivation using a weighted gate that once pushed allowed access to: (1) an area containing both shade (achieved using trees and a shade cloth) and an unshaded, barren area and, (2) a barren area containing no shade. All heifers were tested during the summer months and then again in the fall. We had three predictions: firstly, that heifers would push more weight to enter the ‘with shade’ area on hot summer days compared to when they were provided access during the autumn; secondly, that higher ranked heifers would spend more time under the trees and shade cloth than lower ranked heifers, particularly during the summer months; and, thirdly, that heifers would show reduced motivation (i.e., push less weight) to access the ‘barren’ area.

## 2. Materials and Methods

This study took place during January–April 2017 (summer) and May–June 2017 (autumn), with a total of 58 experimental days, and was carried out at the Federal University of Santa Catarina farm (Florianópolis, Santa Catarina, Brazil; 17°40′25″ S and 48°32′30″ W). The region’s climate is Cfa—a humid subtropical climate according to the Köppen climate classification, as described by [20]. 

### 2.1. Animals, Housing and Management

We used 18 non-pregnant, 42-month old dairy heifers (Holstein and Jersey x Holstein cross-bred) with a mean body weight of 298 kg (SD = 28.3). The heifers were managed using a rotational pasture system [21], and Voisin grazing, as described by [22]; heifers were moved to a new enclosed 50 × 50 m^2^ electric fenced paddock every day at 8:00. There was no shade available in any of the home paddocks. Water was available at all times in 500 L water troughs placed in the home paddock, the holding area and both experimental areas. Mineral saltboxes were also provided ad libitum in the home paddock.

### 2.2. Experimental Areas

**Figure 1:**
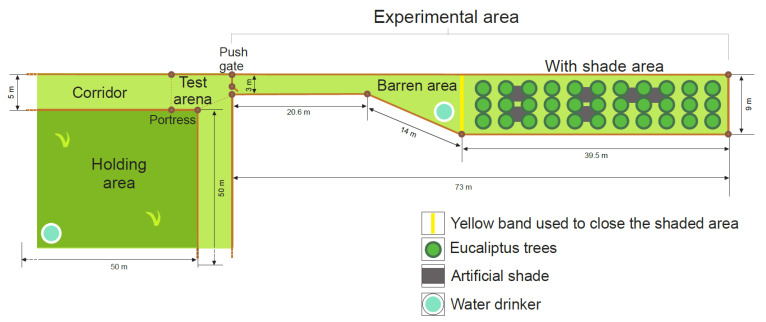
A view of the holding area, the test arena, the push gate and the barren (empty space), and the shaded experimental areas. When tested in the WITH SHADE treatment, heifers (*n* = 18) were allowed access to both the empty space and shaded areas. When tested in the BARREN treatment, heifers were only allowed access to the empty space and were prevented from accessing the shaded area by a yellow fencing material. Heifers were tested during the summer and again in the autumn.

The experimental WITH SHADE area (Figure 1) was an empty space consisting of a barren dirt area that was 140 m^2^, and an adjacent shaded area that was 360 m^2^ (40 m × 9 m) containing *Eucalyptus* sp. trees (~20 m^2^ of shade/animal). Shade consisted of both simple shade (the sun was partially interrupted by the trees and branches) and a composite shaded area (tress plus shade cloth provided 80% protection against solar radiation) that covered approximately 75 m^2^ (25 m × 3 m) of the total shaded area. The shade cloth was positioned at a height of 3 m, between the trees in the shaded area, providing an additional 4 m^2^ of shade/animal. To enter the shaded area, heifers were required to walk through the barren dirt lot area located between the gate and the shade, referred to from hereon in as the BARREN area. When tested in the experimental BARREN treatment, heifers were only allowed access to the barren dirt lot area and were prevented from entering the shaded area using yellow fencing (Figure 1). The entire experimental area was enclosed using electric fencing.

### 2.3. Motivational Testing

We used operant conditioning to test heifers’ motivation to push a weighted gate to access the WITH SHADE and BARREN areas, as described below.

### 2.4. The Gate

A wooden one-way gate, 0.8 m wide and 1.6 m high, fitted with a fixed pulley system that allowed weights (5 kg increments) to be attached was used to test motivation. Force (F) used to push the gate was measured in newtons and kg equivalent. The weight on the gate was increased by 5 kg every 2 days during the testing periods; thus, animals were required to pay a higher price to access the reward as the weight on the gate increased.

### 2.5. Habituation and Training 

Given that our primary objective was to determine whether heifers were motivated to access shade during hot weather, in summer, we only tested heifers on days when the weather was sunny and/or partially cloudy. For the other two conditions (BARREN area and WITH SHADE area in Autumn) the experimental days were not limited by weather.

The heifers were habituated to the experimental set up daily for 3 days, with the session beginning at 12:00. The researchers entered the home paddock and rang a bell to signal to the heifers that they were going to be moved to the holding area directly adjacent to the test arena with the weighted gate. Heifers were then moved individually into the test arena and initially encouraged to pass through the open gate into the BARREN and WITH SHADE areas. Once all the heifers had passed through the gate and were in the BARREN and WITH SHADE areas, they were free to remain there until either some heifers returned voluntarily to the exit gate and were then taken to the home paddock, the weather turned cloudy, or it was 16:00—at which time, they were all gently moved back to their home paddock. The 3-day habituation period ended when all heifers had learned to move to the holding area as a group, and, from there, to the shaded area individually. 

After the habituation period, all the heifers were trained individually for 16 days to push the gate, with access to shade as the reward reinforcing this behaviour. The goal of the training phase was to ensure that all heifers recognized the gate and learned that they could open the gate using their head and body. The method of bringing the animals from the holding area to the experimental area was the same as the one used during the habituation period, with the addition of ringing a bell to signal that the heifer was close to the test arena.

The gate was closed by 15° every 2 ± 1 days, until the heifers learned to push the gate open (i.e., 90°, 75°, 60°, 45°, 30°, 15°, closed). If a heifer did not perform the daily task of passing through the gate within 2 min, she was given a second opportunity later on that session. If a heifer failed to pass through the gate within 2 min on the second attempt, she was gently encouraged to pass through the gate using vocal encouragement and/or gentle rump nudges. If a heifer, again, failed to pass through the gate on the second attempt, the weight on the gate was reduced to that of the previous day when she successfully opened the gate. If, again, the heifer failed to pass through the gate, for ethical reasons she was allowed to pass through the open gate to re-join the group. 

### 2.6. Motivation to Access the WITH SHADE Area in Summer

The sequence of events during the testing period mirrored that of the training phase described above. Each heifer was tested individually. Heifers were initially required to push 5 kg, with an additional 5 kg added after 2 successful days of pushing a given weight; this continued until each heifer failed to push the weighted gate. If a heifer failed to push the gate within 2 min of entering the test arena, she was returned to the holding area and retested at the end of the day’s test session. If the heifer failed to push the weighted gate during 2 min of this second attempt, the gate was opened and she was allowed to enter the shaded area to re-join the group. The last weight successfully pushed by each heifer was considered to be the maximum weight pushed. To control for order of training and testing, the order of testing of the heifers was changed daily.

### 2.7. Motivation to Access BARREN Area in Summer

To ensure that the heifers were aware that the shaded area was not available, yellow fencing material was added near the gate and the electric fence. Heifers were habituated to the new setting by gently moving the group into the barren dry lot area for 10 min. After this, the gate was opened and the heifers were allowed to return to their home paddock; heifers always returned immediately once the gate was opened. After 11 d of habituation, heifers’ training was reinforced for 3 days as follows: day 1, the gate was opened at 90° to allow all heifers to enter the empty space without having to push the weighted gate; day 2, the angle of the gate was reduced to 30°; day 3, the gate was closed and heifers were required to push the gate open to access the empty space. The training protocol was the same as described above. 

### 2.8. Motivation to Access the WITH SHADE Area in Autumn

The same 18 heifers were habituated to the same experimental areas (see Figure 1) for 4 d. Training was then reinforced for 3 consecutive days, with the gate open at 90°, 30° and closed (locked), respectively. After the retraining period was completed, the same methodology for testing during the summer described above was employed. The testing phase finished when the heifers refused to enter the WITH SHADE area for more than one hour for three consecutive days. 

### 2.9. Motivation to Access the BARREN Area in Autumn

Given the reluctance of the heifers to work for the WITH SHADE area in autumn we did not test for motivation to access the BARREN area during this season.

### 2.10. Behavioural Measures

The following information was collected during each phase of the experiment (summer and autumn): the order that heifers left the holding area; latency to push the weighted gate; the time(s) from when she entered the test arena to when her body had completely passed through the weighted gate; if the heifer pushed the gate in 2 min (yes/no); whether each heifer took one or two attempts to push the gate. 

We recorded the location within the WITH SHADE area (barren area, under the trees plus cloth shade, or under the tree shade) using 10 min scan sampling. The scans were only made after all heifers were inside the experimental area, after all had pushed the gate.

### 2.11. Social Rank

The social hierarchy of the individuals within the group of heifers was previously determined (see [23]) and was used to assign individuals to one of three social ranks: dominant, intermediate, and subordinate. Briefly, the group of heifers (*n* = 18) were observed twice weekly during their daily offering of 2 kg/animal/day of a commercial ration for cattle (12% CP). The supplement was placed along the ground, adjacent to the fence line, and all agonistic interactions were continuously recorded for an hour, using instantaneous scan sampling with a 2 min interval. This was done twice a week for 20 non-consecutive days. 

### 2.12. Climatic Measures

Ambient temperature and relative humidity measures were collected during the habituation, training, and testing periods. To avoid the heifers licking the temperature loggers, we placed one digital thermo-hygrometer (Incoterm 7664.01.0.00, China) approximately 0.5 m above the ground, immediately adjacent to the barren area, and the second approximately 0.5 m above the ground, under the shaded tree area, immediately adjacent to the fenced area. These data were used to calculate the heat index [24]. Apparent temperature was calculated using the average temperature and relative humidity for the entire experimental period obtained from the Centre of Information of Environmental Resources and Hydrometeorology of Santa Catarina (CIRAM, http://ciram.epagri.sc.gov.br (accessed on 10 June 2017)) that was based on National Weather Service’s (NWS) equation. 

### 2.13. Statistical Analysis 

Descriptive statistics (means and percentages) were provided, summarizing the climatic data. The frequency of individual animal location was registered as scans. The number of scans recorded for each heifer was then divided by the total time (in min) of observation.

Student paired t-tests were used to test differences in climatic measures (maximum temperature and heat index) between summer and autumn and maximum weight pushed, latency to push the gate, duration of training, and test period. To test for associations between social rank and the type of shade used, we chose the locations of the six high ranked heifers and six low ranked heifers, and applied a linear regression. Pearson correlation coefficients were used to test for correlations between maximum weights pushed and body weight. Given that the behaviour measures taken in the WITH SHADE areas were done on a single group, they are not independent, meaning the time spent under sun and shade (the trees plus cloth shade + simple tree shade) is presented descriptively, as means and standard deviations. The analyses were performed in R 2017 [25]. The significance level was set to *p* < 0.05.

## 3. Results

### 3.1. Climatic Data

The maximum temperatures and the heat index (in the direct sun and under the shaded areas) were higher in summer compared to autumn (Table 1). The maximum temperature and the calculated heat index were also higher in summer than in autumn (Table 1). In summer, the lowest maximum temperature in the sun was 34.4 °C (varying from 34.3 to 48.3 °C) and, in autumn, this varied from 22.1 to 37.2 °C.

### 3.2. Motivation to Access the WITH SHADE Area

**Figure 2:**
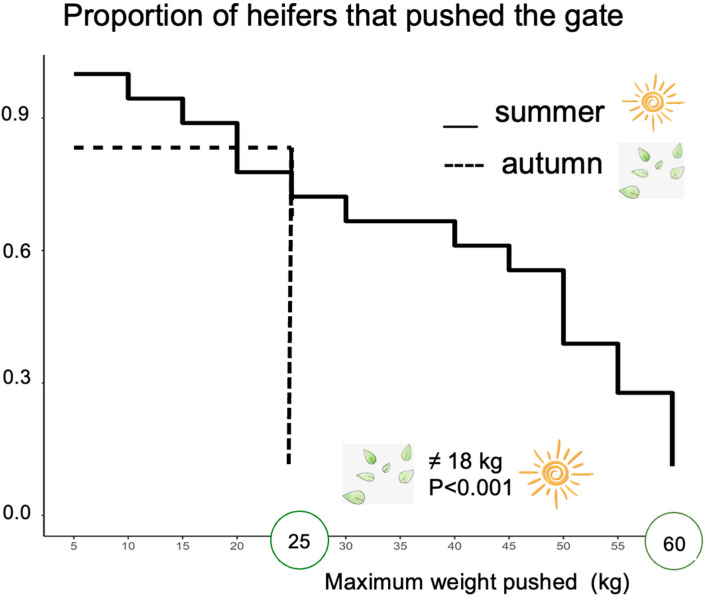
A step graphic for pasture housed heifers’ (*n* = 18) willingness to work (maximum weight pushed) for access to an area with an open dry lot area and a shaded area during the summer (solid line) vs. autumn (dashed line). Maximum weight pushed to access shade was stronger in the summer compared to the autumn (*p* < 0.01).

Once trained, the latency to push the gate did not differ between summer (61 s, SE = 8.6) and autumn (89 s, SE = 17.8; paired *t*-test, *p* > 0.01). On average, heifers pushed 38 kg (SD = 18.3) to access the WITH SHADE area during the summer (range 5 kg to 60 kg). In the autumn, three heifers pushed 0 kg and one heifer pushed a maximum weight of 25 kg; overall, the average weight pushed declined to 22 kg (SD = 6.3) (*p* < 0.001; Figure 2).

There was no relationship between the maximum weight pushed and the individual BW (Pearson correlation, summer: *p* > 0.1 and autumn: *p* > 0.1), or social rank (linear regression, summer: *p* = 0.94 and autumn: *p* = 0.54). The latency to push the gate was inversely related to the maximum weight pushed in both seasons (paired *t*-test, summer: *p* < 0.05 and autumn: *p* < 0.01).

### 3.3. Motivation to Access the BARREN Area

Once habituated to the barren environment, all heifers refused to engage in the operant task of pushing the weighted gate to access this environment.

### 3.4. Social Rank and Shade Use

There was an association between dominance and shade use in summer, but not in autumn; in summer, the low social ranked heifers used the trees plus cloth shade less (39%, SE = 5.9), compared to the high rank heifers (66.5%, SE = 8.5) (linear regression, *p* < 0.05). Additionally, the low ranked heifers used the tree shade more (39.2%, SE = 5.35) than the high ranked heifers (19.2%, SE = 6.43) (linear regression, *p* = 0.01).

### 3.5. Behaviour 

In summer, heifers stayed in the WITH SHADE area between 60 min and 215 min per day—on average, 137 min/d (SD = 36 min). In autumn, they stayed in this area between 25 min and 150 min per day—on average, 88 min/day (SD = 50 min). In summer, heifers spent 52.6% (SE = 5.1) of the available time under the tree shade with the cloth, and 27.4% of the time (SE = 3.5) under the tree shade. In autumn, they spent 25.1% (SE = 3.4) of the time under the tree shade and 15.2% (SE = 3.4) under the trees plus cloth shade.

## 4. Discussion

In the current study, heifers that were continuously housed on pasture pushed more weight to access the WITH SHADE area on hot summer days than they did in autumn, when temperatures were cooler. The maximum weight pushed to open the gate was inversely related to the latency to push the gate in both seasons. In other words, more motivated heifers not only pushed more weight, but completed the task faster on hot summer days. The warmer temperatures during the summer likely caused the heifers to spend more time in the shaded areas of the enclosure in summer than in autumn. Regardless of the season, the maximum temperatures and heat index were higher in the areas of direct sunlight, compared to the shaded areas; the shade provided a cooler environment for the animals. Our results are the first to show that shade is a valuable resource for dairy heifers that are continuously managed in a pasture-based system, particularly during the warmer summer months. 

There is a body of evidence indicating that animals are willing to work for access to a given reward until, at some point, likely when the demand price becomes too high, they stop working for access [26]. Fraser and Duncan [27] suggest that animals have “motivational affective states” and will work to access resources that are pleasurable if the cost is sufficiently low. During the present study, the heifers were presented with a trade-off between grazing and staying cooler. By design, the heifers were moved every morning to a new paddock with fresh pasture, and the holding area adjacent to the gate was almost void of pasture, likely making this area less attractive. The reduced heat index recorded during the autumn (compared to the summer) likely explains why the heifers were less motivated to access the WITH SHADE area during the autumn. The cooler temperatures also likely increased the motivation for the heifers to graze during the early afternoon hours, compared to during the hot summer. Unfortunately, our study was not designed to provide any insight into the motivation to graze versus seeking the cooler microclimate provided by the shaded area. We encourage future research to disentangle these two contrasting motivations.

The fact that heifers pushed some weight to access the shade in autumn suggests that they still valued accessing the shade, albeit to a far lesser degree than in the summer. Whether this was driven by the cooler environmental conditions under the shade, compared to being in full sun, or due to the lower pasture quality in their home paddock is not known. However, there is some evidence that cows enjoy natural shade. In a study carried out in summertime, comparing natural shade and an artificial shelter with similar climate conditions, cattle preferred to stay under the natural shade [28].

When provided the opportunity to access only the BARREN area, heifers chose not to work, providing some evidence that they were not simply seeking additional space. This finding is supported by the work of McConnachie et al. [18], who reported that free stall housed dairy cattle given the opportunity to work for access to an empty space were far less motivated compared to when the empty space contained a mechanical brush. Failure to work to access the empty space may be explained by the heifers showing avoidance behaviour for the empty space [29]. Although we did not evaluate the preference for each of the specific areas in the WITH SHADE treatment (see [6]), our work suggests that motivation is also associated with preference, given that, under the conditions of the present study, heifers showed some preference for the shaded areas, particularly during the warm summer months.

In both summer and autumn, it was cooler under the shade compared to the non-shaded areas in the WITH SHADE experimental area. In summer, the higher ranked heifers spent the most time in the shaded areas compared to the lower ranked animals. Unfortunately, we were unable to measure the climatic differences under the two types of shade provided within this treatment. However, the fact that the higher ranked heifers spent more time under the trees plus shade cloth area compared to the tree shaded area (and low ranked heifers spent more time in the treed area) suggests that there were aspects of the trees plus shade cloth microenvironment that were important to the high ranked heifers. High ranking individuals have been shown to have priority access to resources over low ranking individuals [30,31]. Schütz et al. [32] concluded that dairy cows have preferences for shade that offers greater protection against solar radiation in the summer. As social hierarchy was not correlated with the weight pushed to access shade, we speculate that some of the low ranking heifers had similar motivation to access the shade as their dominant counterparts did, but were unable to secure the shade due to social competition. 

The maximum weight pushed by the heifers in summer equated to approximately 20% of their body weight, which is similar to other studies. Lactating Canadian Holstein cows pushed, on average, a maximum of 11% of their body weight [17]; Angus–Hereford cross beef heifers pushed 18% of their body weight [16]; Holstein–Friesian cows pushed 40% of their body weight when deprived of being able to lie down [33]. Given their reduced metabolic demands, heifers likely suffer less from the effects of heat stress [34]. However, our work suggests that, even when not lactating, shade is an important resource for dairy heifers.

We noted no relationship between the maximum weight pushed to access shade in summer and heifers’ body weight (see [14]). Tucker et al. [33] also found no difference between the body weight of cows that pushed or did not push, concluding that there is individual variation in motivational states. This, and other studies, suggests that motivation is an individual characteristic [29,35]. As pointed out by Van Os et al. [16], individual personality may play a role in motivation. Personality in animals [36] can be defined as “those characteristics of individuals that describe and account for temporally stable patterns of affect, cognition, and behaviour”. Personality is focused on behavioural traits [36], meaning that individual differences, such as the difference identified in motivation to access shade, are worthy of future work. For example, characteristics like fearfulness, sociability, exploratory behaviour, or response to novelty [37,38] could differently influence the individual motivation of a heifer to push the gate to access shade.

The amount of available artificial shade (4 m^2^/shade/heifer) in the present study was within the range of 2.4 and 9.6 m^2^/shade/cow that was tested by Schütz et al. [3]. The latter authors found that agonistic interactions were inversely related to the amount of shade available, particularly when the heat load increased. Given that cows will actively compete for highly valued resources [39], we suggest that the amount of shade provided in our study was likely insufficient for the size of the group, although more work is needed to confirm this. Tucker et al. [40] and Coimbra et al. [41] both comment on the importance of offering resources in ways that allow all animals within a herd to access them simultaneously.

We also encourage future research to investigate the motivation of dairy cattle to access shade for other reasons (for example, elements that cattle may desire to have access to, such as trees for grooming) and investigate the influence of cows’ personality in motivation tests.

## 5. Conclusions

The heifers used in the current study, managed in a pasture-based system, were motivated to access a shaded area, particularly during the hot summer. As expected, motivation was stronger in the summer than in autumn, and was positively associated with the latency to pass through the gate. Additionally, heifers were not motivated to push the gate to access the barren area. High ranked heifers chose to spend the majority of their time in the shade, specifically under the trees plus cloth shade, whereas the lower ranking heifers spent most of their time under simple tree shade.

## Figures and Tables

**Table 1:** Differences between maximum temperature (°C) and heat index (°C) in summer and autumn in the direct sunlight (BARREN) and in the shaded area (WITH SHADE) (mean ± SE) in Santa Catarina, Brazil.

Season	Max. T BARREN	Max. T WITH SHADE	*p*-Value	Heat Index BARREN	Heat Index WITH SHADE	*p*-Value
Summer	41.3 ± 0.45	34.0 ± 0.45	0.001	43.4 ± 0.71	36.0 ± 0.70	0.001
Autumn	30.3 ± 1.07	25.4 ± 0.77	0.001	28.1 ± 1.05	24.4 ± 0.78	0.01

## Data Availability

The following are available online at dx.doi.org/10.6084/m9.figshare.16441254, original data Shade_R.xlxs.
